# Research Trends and Most Influential Clinical Studies on Anti-PD1/PDL1 Immunotherapy for Cancers: A Bibliometric Analysis

**DOI:** 10.3389/fimmu.2022.862084

**Published:** 2022-04-11

**Authors:** Yanhao Liu, Yan Xu, Xi Cheng, Yaru Lin, Shu Jiang, Haiming Yu, Zhen Zhang, Linlin Lu, Xiaotao Zhang

**Affiliations:** Department of Radiation Oncology, The Affiliated Qingdao Central Hospital of Qingdao University, Qingdao, China

**Keywords:** bibliometric analysis, lung cancer, PD1/PDL1, clinical trials, melanoma, immunotherapy, tumor mutation burden

## Abstract

In this study, a bibliometric analysis was carried out to identify the most influential clinical studies and research trends on anti-programmed cell death 1/programmed cell death 1 ligand 1 (anti-PD1/PDL1) immunotherapy. On January 1, 2022, we used Web of Science to identify the 100 most frequently cited papers on clinical studies investigating anti-PD1/PDL1 immunotherapy, and extracted the following data: publication year, source title, country/region, institution, and the total number of citations. The research design and area were classified independently by the authors. Subsequently, we carried out a bibliometric analysis to determine the trends and identify the major journals on anti-PD1/PDL1 immunotherapy. The authors analyzed the current research hotspots based on papers published in major journals from 2020 to 2021. These 100 papers were cited a total of 138,840 times, and the median number of citations was 899.5 (range: 341–7,983). “Safety, activity, and immune correlates of anti-PD-1 antibody in cancer” by Topalian et al. had the highest number of citations (7,983 times). *New England Journal of Medicine* had the highest number of top-cited papers (40 papers), average citations per paper (1,558.3 citations), and rate of top-cited papers (65.6%). Authors from the USA contributed most of the papers (76 papers). Lung cancer (30 papers, 46,422 citations) and melanoma (20 papers, 30,881 citations) were the most cited research areas. In summary, anti-PD1/PDL1 has become standard treatment for various cancer, while adjuvant anti-PD1/PDL1 therapy is currently a research hotspot. *New England Journal of Medicine* was identified as the most influential journal in this area. Non-small cell lung cancer and melanoma are the most well-studied cancers, while nivolumab and pembrolizumab are the most commonly investigated anti-PD1/PDL1 antibodies. Further studies are warranted to identify effective predictive biomarkers or models, clarify the molecular mechanism of combined therapy, and establish optimal therapeutic strategies. This study may assist researchers in obtaining a comprehensive impression of the landscape and current trends in anti-PD1/PDL1 immunotherapy and gain inspiration to conduct further studies.

## Introduction

Cancer has become one of the most health-threatening diseases due to the modern lifestyle and prolonged life expectancy of individuals. Despite advances in research, the prognosis of patients with cancer (particularly late-stage disease) is disappointing. In recent years, the use of immune checkpoint inhibitors (ICIs) has further optimized the treatment patterns for multiple types of cancers. Programmed cell death 1 (PD1): programmed cell death 1 ligand 1 (PDL1), and cytotoxic T lymphocyte-associated protein 4 (CTLA4): B-lymphocyte activation antigen B7 (B7-1; also termed CD80) are the most extensively studied immune checkpoints for cancer immunotherapy.

PDL1 and CTLA4 are mainly expressed on the cytomembrane of immune cells, while PD1 and B7-1 are mainly expressed on the cytomembrane of antigen-presenting cells. The PD1:PDL1 and CTLA4:B7-1 interactions inhibit T cells, thereby modulating cytokine release and cell proliferation, and affecting the cell phenotype (1). Some tumors express PDL1, which contributes to immune evasion by inhibiting cytotoxic responses. Thus, anti-PD1/PDL1 antibodies can promote T cell activation and enhance anti-tumor immunity by blocking the PD1:PDL1 interaction (2). The mechanisms involved in anti-CTLA4 immunotherapy are complex. The classic theory suggests that blocking CTLA4:B7-1/B7-2 interactions enhances CD28 co-stimulation, thus promoting T cell activation. Given that B7-1 and B7-2 are only expressed on APCs, CTLA4 blockade is thought to mainly occur in tumor-draining lymph nodes, where APCs present tumor antigens to prime naive T cells (3). Nevertheless, new studies suggest that CTLA4 blockade within the tumor microenvironment could decrease the activation threshold of T cells, while selectively depleting immunosuppressive regulatory T cells; these effects increase the number of tumor-specific CD8 T cells (4–6). Additionally, recent studies demonstrated the involvement of PDL1:B7-1 cis-interaction in anti-PD1/PDL1 and anti-CTLA4 immunotherapy, which may reveal new mechanisms and further optimize the therapeutic strategy (1).

Ipilimumab, as the most widely used anti-CTLA4 antibody, has been applied to the treatment of advanced melanoma since the 2000s (7). However, anti-PD1/PDL1 antibodies showed superior tolerability and efficacy than ipilimumab for the treatment of melanoma and improved the prognosis of patients with other cancers (8, 9). Anti-PD1/PDL1 immunotherapy for cancers is a burgeoning area of research, with >10,000 clinical studies published in the last decade. Therefore, it is challenging for researchers to identify the most influential studies, master the overall landscape, or stay informed regarding research trends. Thus, a systematic and quantified analysis is warranted to provide comprehensive knowledge to researchers.

Classic reviews usually present the advances in a particular area of research and are limited by the experience and perspective of the authors. In contrast, a bibliometric analysis can objectively, comprehensively, and quantitatively evaluate a whole academic discipline based on the information and a citation network of the relevant publications (10). The visualization based on the bibliometric analysis (i.e., bibliographic coupling) can present the results in multiple forms, which are intuitive and comprehensible for readers (11). Moreover, publications that are important nodes in the citation network can be recognized, and benefit the authors in discussing the history and current status of the research area.

Some previous bibliometric analyses focused on anti-PD1/PDL1 immunotherapy; however, all these analyses were characterized by limitations. A recently published bibliometric analysis including molecular mechanisms, randomized clinical trials, and meta-analysis of PD1/PDL1 provided an overview of this area (12). However, many frequently cited important randomized clinical trials were not included in this analysis (8, 13–17). Other bibliometric analyses only performed cursory searches and did not distinguish between clinical studies, reviews, and basic research (18, 19). All these studies only performed basic analyses and listed superficial information, but lacked intuitive visualization and in-depth discussion. Therefore, it is necessary to conduct a more rigorous, profound, and helpful bibliometric analysis.

This bibliometric analysis focused on published clinical studies of anti-PD1/PDL1 immunotherapy, and aimed to assist researchers in obtaining a comprehensive impression of the landscape as well as current trends in this area. This study identified the 100 most frequently cited papers to present the historical development and research hotspots. Moreover, the present analysis only included clinical studies on anti-PD1/PDL1 immunotherapy, thus improving the comparability between papers. The authors rigorously designed the search strategy and independently reviewed the search results, to ensure that important papers were not omitted. The search was conducted on January 1, 2022, approximately 2 years after the latest similar study (12). Furthermore, an additional bibliometric analysis based on the latest major publications was conducted to determine the newest research hotspots.

This study answered the following research questions of anti-PD1/PDL1 immunotherapy: 1) Which are the most influential clinical studies and top contributing journals? 2) Which are the top contributing countries, institutions, and authors? 3) Which are the most studied cancers and drugs? 4) What are the historical research trends, current research status, and future research directions? Based on these results, researchers can not only identify key articles, journals, and potential collaborators, but also gain inspiration from the research trends herein to conduct further studies.

## Methods

### Study Selection

We used Web of Science (Science Citation Indexing Expanded database) to identify the most frequently cited papers. The reason for using this database is that it is the most frequently used and acceptable database for scientific or bibliometric studies, includes nearly all of the influential and high-quality journals, and contains comprehensive citation index records (20). In addition, a previous study indicated that the document type labels of Web of Science are more accurate than other databases such as Scopus (21). We conducted a literature search for original research articles without restrictions on language and time of publication. The authors designed the search strategy by considering various writing formats and conducted multiple tests to ensure preciseness. The detailed search strategy is presented in **Supplementary Material S1**. Subsequently, for the identification of the 100 most frequently cited clinical studies on anti-PD1/PDL1 immunotherapy, the authors ranked the search results according to the number of citations and reviewed the search results to eliminate irrelevant papers. Next, we used Web of Science to extract the following information: time of publication; journal; country/region; institution; the total number of citations; and average number of citations per year (i.e., the number of citations per month × 12). The journals which published top-cited papers were identified, the newest papers regarding clinical studies on anti-PD1/PDL1 immunotherapy in these journals were reviewed, and data for all papers published in these journals since 2020 were extracted for analysis to evaluate recent research hotspots.

The type of cancer, type of anti-PD1/PDL1 antibody, and research design of the 100 identified articles were independently categorized by two authors who read the abstract and, if necessary, the whole paper. In case of discrepancy concerning a particular paper, a senior researcher read the paper and reached a final decision.

### Statistical Analysis

The Excel software (Microsoft, Redmond, WA, USA) was used for descriptive statistical analysis and to produce diagrams. The “bibliometrix” and “circlize” packages of R software (v4.1.1) were used for bibliometric analysis and data visualization. An online platform (https://bibliometric.com) and VOSviewer software (v1.6.17) were used to construct a bibliographic coupling network based on the relationship between journal, country, co-authors, and keywords, as well as for network visualization and analysis.

## Results

This bibliometric analysis is conducted to reveal (1) the most influential clinical studies, the top contributing (2) journals, (3) countries, (4) institutions, and (4) authors, the most studied (5) cancers, and (6) drugs, and (7) the research hotspots of anti-PD1/PDL1 immunotherapy. The literature search yielded 50,412 articles. The 100 most frequently cited papers are listed in **Supplementary Table S1**. The total number of citations for these 100 papers was 138,840, and the median number of citations was 899.5 (range: 341–7983). “Safety, activity, and immune correlates of anti-PD-1 antibody in cancer” by Topalian et al. had the highest number of total citations (7,983 citations) and the third-highest average number of citations per year (833.0 citations) (9). “Pembrolizumab versus chemotherapy for PD-L1-positive non-small-cell lung cancer” by Brahmer et al. had the highest average number of citations per year (941.8 citations) and the fifth-highest number of total citations (4,866 citations) (14). “Atezolizumab plus bevacizumab in unresectable hepatocellular cancer” by Theelen et al., published in May 2020, was the most recent publication among the 100 most cited papers (22). The top 10 cited papers (**Table 1**) were all published in *New England Journal of Medicine* (*N. Engl. J. Med.*). These papers included one prospective study, two phase 1 clinical trials, one phase 2 clinical trial, and six randomized phase 3 clinical trials. The historical direct citation network among the top-cited papers is shown in **Supplementary Figure S1**.

**Table 1:** The 10 most cited papers on anti-PD1/PDL1 immunotherapy for cancers.^a^

Rank	Title	Corresponding author	Year	Total citations	Average citations per year (rank)
1	Safety, Activity, and Immune Correlates of Anti-PD-1 Antibody in Cancer	Topalian, SL	2012	7,983	833.0 (3)
2	Nivolumab versus Docetaxel in Advanced Nonsquamous Non-Small-Cell Lung Cancer	Borghaei, H	2015	5,521	883.4 (2)
3	Safety and Activity of Anti-PD-L1 Antibody in Patients with Advanced Cancer	Brahmer, JR	2012	5,052	527.2 (11)
4	PD-1 Blockade in Tumors with Mismatch-Repair Deficiency	Diaz, LA	2015	4,938	750.1 (4)
5	Pembrolizumab versus Chemotherapy for PD-L1-Positive Non-Small-Cell Lung Cancer	Brahmer, JR	2016	4,866	941.8 (1)
6	Combined Nivolumab and Ipilimumab or Monotherapy in Untreated Melanoma	Hodi, FS	2015	4,791	737.1 (5)
7	Nivolumab versus Docetaxel in Advanced Squamous-Cell Non-Small-Cell Lung Cancer	Brahmer, J	2015	4,405	677.7 (6)
8	Pembrolizumab for the Treatment of Non-Small-Cell Lung Cancer	Garon, EB	2015	3,618	542.7 (10)
9	Nivolumab versus Everolimus in Advanced Renal-Cell Cancer	Motzer, RJ	2015	3,453	559.9 (9)
10	Nivolumab in Previously Untreated Melanoma without BRAF Mutation	Robert, C	2015	3,444	492.0 (14)

a These 10 papers were published in the New England Journal of Medicine.

PD1, programmed cell death 1; PDL1, programmed cell death 1 ligand 1.

### Time of Publication and Research Design

**Figure 1:**
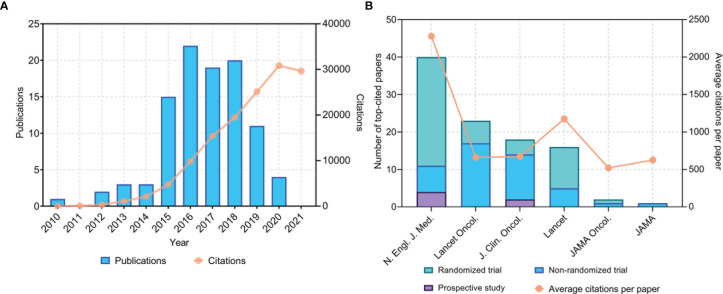
**(A)** Time of publication and distribution of citations of the 100 most cited papers on anti-PD1/PDL1 immunotherapy. **(B)** Total number of top-cited papers and average citations per paper in major journals. PD1, programmed cell death 1; PDL1, programmed cell death 1 ligand 1. N. *Engl. J. Med.*, *New England Journal of Medicine*; *JAMA*, *Journal of the American Medical Association*; *JAMA Oncol.*, *JAMA Oncology*; *J. Clin. Oncol.*, *Journal of Clinical Oncology*; *Lancet Oncol.*, *Lancet Oncology*.

The 100 most frequently cited papers were published between 2010 and 2020 (**Figure 1A**). Most of these top-cited papers (87 papers) were published between 2015 and 2019. The year with the highest number of published papers (of the top 100 articles) was 2016 (22 papers). The papers included six prospective studies, 19 phase 1 clinical trials, four phase 1/2 clinical trials, 26 phase 2 clinical trials, one randomized phase 2/3 clinical trials, and 43 randomized phase 3 clinical trials. Of the 26 phase 2 clinical trials, 19 were non-randomized and nine were randomized. Most of these studies (94 papers) focused on anti-PD1/PDL1 antibodies without local treatment for advanced, metastatic, or recurrent cancers, while the remaining six studies evaluated anti-PD1/PDL1 antibodies as an adjuvant treatment before or after radical resection or chemoradiotherapy for cancers. Among the 94 studies, the anti-PD1/PDL1 antibodies were used as first-line monotherapy (12 studies), part of combined first-line therapy (25 studies), second-line or salvage monotherapy (54 studies), and part of combined second-line or salvage therapy (three studies). Of the 100 studies, 48 included control groups. Among these studies, 27 compared anti-PD1/PDL1 antibodies with chemotherapy, and 10 compared anti-PD1/PDL1 antibodies with ipilimumab.

### Journals

The 100 most cited papers were published only in six major journals (**Figure 1B**). *N. Engl. J. Med.* published the largest number of papers (40 papers), followed by *Lancet Oncology* (23 papers), *Journal of Clinical Oncology* (18 papers), and *Lancet* (16 papers). The journal with the highest average number of citations per paper was *N. Engl. J. Med.* (2,278 citations). It is worth mentioning that the 11 most cited clinical studies on anti-PD1/PDL1 immunotherapy were all published in *N. Engl. J. Med*.

The citation analysis of papers on anti-PD1/PDL1 immunotherapy published in these journals between 2010 and 2020 is shown in **Table 2**. A total of 488 papers were published in these journals. Among these journals, the average rate for the publication of top-cited papers was 20.5%. *N. Engl. J. Med.* had the highest average citations per paper (1,558.3 citations) and rate for the publication of top-cited papers (65.6%), followed by *Lancet*.

**Table 2:** Citation analysis of papers on anti-PD1/PDL1 immunotherapy published in major journals between 2010 and 2020.

Journal	Total number of top-cited papers	Total number of papers	Average number of citations per paper	H-index	Percentage of top-cited papers
*N. Engl. J. Med.*	40	61	1,558.3	51	65.6%
*Lancet Oncol.*	23	130	200.9	73	17.7%
*J. Clin. Oncol.*	18	164	171.6	83	11.0%
*Lancet*	16	30	683.9	28	53.3%
*JAMA Oncol.*	2	95	100.4	53	2.1%
*JAMA*	1	3	259	3	33.3%
Total	100	488	369.2	176	20.5%

PD1, programmed cell death 1; PDL1, programmed cell death 1 ligand 1; H-index, Hirsch-index; Engl. J. Med., New England Journal of Medicine; JAMA, Journal of the American Medical Association; JAMA Oncol., JAMA Oncology; J. Clin. Oncol., Journal of Clinical Oncology; Lancet Oncol., Lancet Oncology.

### Countries and Institutions

**Figure 2:**
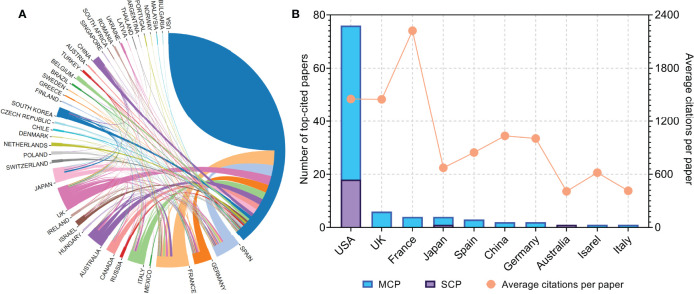
**(A)** Network visualization map of inter-country/-regional collaboration. **(B)** Total number of top-cited papers and average citations per paper according to the countries of corresponding authors. MCP, multiple-country publications; SCP, single-country publications.

**Figure 3:**
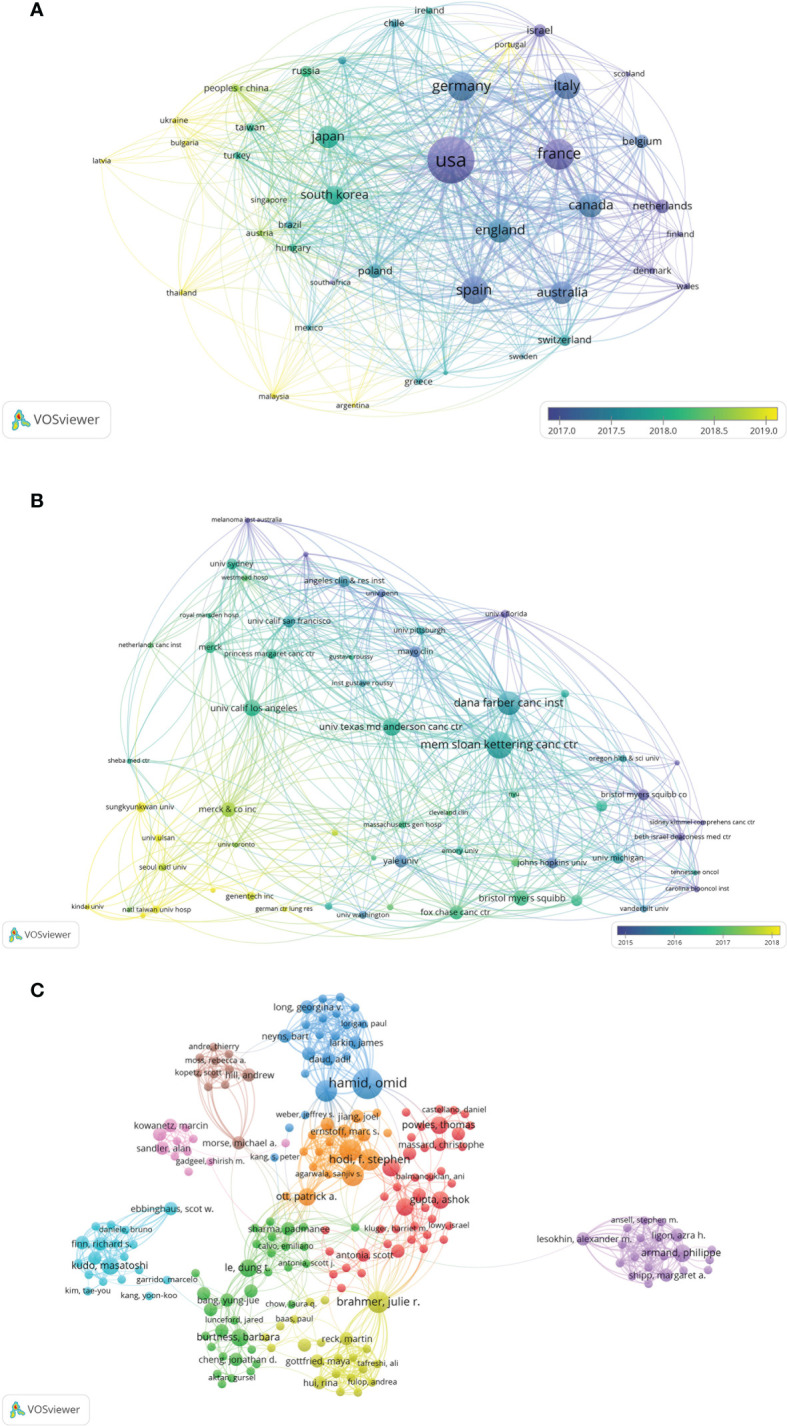
**(A)** Network visualization of the countries/regions for the 100 most cited papers according to the average year of publication. **(B)** Network visualization of institutions for the 100 most cited papers according to the average year of publication. **(C)** Network visualization of authors with at least 2 of the 100 most cited papers according to cluster analysis. The circle size represents the number of articles included in the 100 most cited articles; the width of the curved line represents the strength of the connection; and the distance between two nodes approximates the relatedness.

The authors of the 100 top-cited papers represented 40 countries or regions (**Figure 2A**), while the corresponding authors were from 10 countries or regions (**Figure 2B**). The corresponding authors from the USA contributed most of the papers (76 papers). Corresponding authors from France had the highest average number of citations per paper (2,222 citations). The network visualization maps for collaborations between countries/regions and institutions are shown in **Figures 3A, B**, respectively. Developed countries were at the center of the network, producing more publications than developing countries. Studies contributed by South American or Asian countries were mainly published more recently than those from other regions. The Memorial Sloan-Kettering Cancer Center was the research institution with the most published papers (58 papers), followed by the University of Texas MD Anderson Cancer Center (42 papers), and the Dana Farber Cancer Institute (39 papers).

### Authors

A network and clustering analysis of the coauthors of the 100 most frequently cited papers was constructed (**Figure 3C**). The authors were classified into different clusters mainly according to the country, institution, and research area. Researchers with at least eight publications are listed in **Table 3**. The most prolific author was Brahmer JR (12 papers), followed by Hamid O (11 papers), and Hodi FS (11 papers). Papers published by Brahmer JR had the highest total number of citations (26,417 citations). Among the corresponding authors of these papers, Hodi FS, Motzer RJ, and Topalian SL had the highest corresponding author frequency (four times).

**Table 3:** Authors of at least eight of the top-cited papers on anti-PD1/PDL1 immunotherapy for cancers.

Name	Total number of most cited papers	Total number of citations of most cited papers	Average number of citations per paper	Corresponding author frequency	Articles fractionalized^a^
Brahmer JR	12	26,417	2,201.4	3	0.59
Hamid O	11	16,794	1,526.77	0	0.55
Hodi FS	11	22,332	2,030.2	4	0.50
Horn L	10	22,482	2,248.2	2	0.44
Wolchok JD	10	18,046	1,804.6	2	0.44
Paz-Ares L	9	17,045	1,893. 9	2	0.34
Reck M	9	17,074	1,897.1	2	0.43
Robert C	9	15,356	1,706.2	3	0.40
Ascierto PA	8	14,242	1,780.3	0	0.30
Larkin J	8	16,734	2,091.8	1	0.33
Pardoll DM	8	23,528	2,941	1	0.33
Powles T	8	7,376	922	2	0.41

a Articles fractionalized = paper number/total number of authors of the papers.

### Cancers and Anti-PD1/PDL1 Antibodies

**Figure 4:**
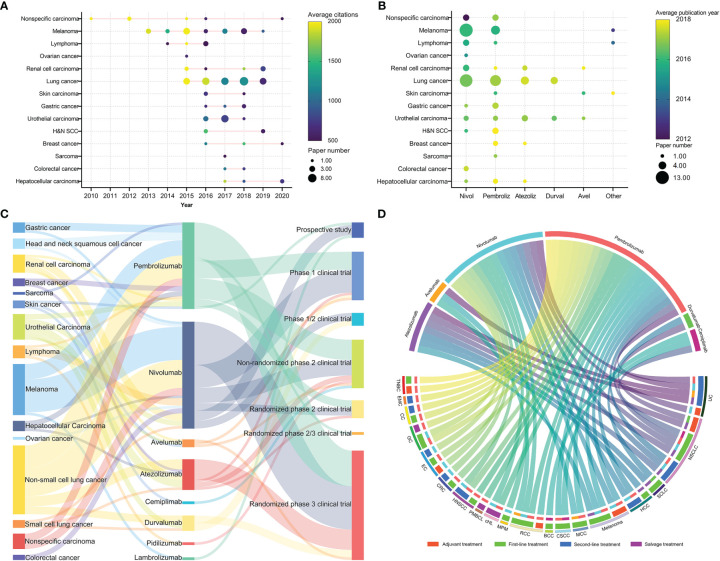
**(A)** Distribution of publication time and citations of papers on cancers. The node size represents the number of publications; the color represents the average citations of the papers. **(B)** The relationship between cancers and anti-programmed cell death 1/programmed cell death 1 ligand 1 (anti-PD1/PDL1) antibodies. The node size represents the number of publications; the color represents the average year of publication. Atezoliz, atezolizumab; Avel, avelumab; Durval, durvalumab; H&N SCC, head and neck squamous cell cancer; Nivol, nivolumab; Other, lambrolizumab, cemiplimab, or pidilizumab; Pembroliz, pembrolizumab. **(C)** The relationship between cancers, anti-PD1/PDL1 antibodies, and research types for the 100 most cited papers. **(D)** Chordal graph of approved indications of anti-PD1/PDL1 antibodies. BCC, basal cell cancer; CC, cervical cancer; cHL, classical Hodgkin lymphoma; CRC, colorectal cancer; CSCC, cutaneous squamous cell cancer; EC, esophageal cancer; EMC, endometrial cancer; GC, gastric cancer; HCC, hepatocellular cancer; HNSCC, head and neck squamous cell cancer; MCC, Merkel cell cancer; MPM, malignant pleural mesothelioma; NSCLC, non-small cell lung cancer; PMBCL, primary mediastinal large B-cell lymphoma; RCC, renal cell cancer; SCLC, small-cell lung cancer; TNBC, triple-negative breast cancer; UC, urothelial cancer.

The cancers investigated in the 100 most cited papers are shown in **Table 4**. Half of these papers pertained to lung cancer (30 papers, 46,422 citations) or melanoma (20 papers, 30,881 citations). The average citations per paper (3,494 citations) and average citations per year per paper (411.8 citations) of non-specific cancer (six papers) were higher than those of other cancers. The research and citation trend for different cancers is shown in **Figure 4A**. Studies on non-specific cancers were initiated earlier than those on specific cancers and were highly cited. The three papers on non-specific cancer published after 2012 focused on pembrolizumab for brain metastasis or tumors with defective DNA mismatch repair (dMMR)/high microsatellite instability (MSI-H). Since 2013, the number of studies on specific cancers has increased considerably.

**Table 4:** Types of cancers investigated in the top-cited papers on anti-PD1/PDL1 immunotherapy.

Cancer type	Number of papers	Total number of citations	Average number of citations (per paper)		Average number of citations per year (per paper)
Lung cancer	30	46,422	1,547.4	336.5	
Melanoma	20	30,881	1,544.1	266.6	
Urothelial cancer	10	8,484	848.4	173.4	
Renal cell cancer	7	8,692	1,241.7	294.5	
Non-specific cancer	6	21,063	3,510.5	441.1	
Lymphoma	5	4,218	843.6	132.6	
Gastric cancer	4	2,740	685	167.1	
Head and neck squamous cell cancer	4	4,241	1,060.3	260.1	
Hepatocellular cancer	4	4,177	1,044.3	370.3	
Skin cancer	3	1,938	646	136.6	
Breast cancer	3	3,077	1,025.7	300.4	
Colorectal cancer	2	1,873	936.5	227.7	
Ovarian cancer	1	609	609	89	
Sarcoma	1	425	425	88	

PD1, programmed cell death 1; PDL1, programmed cell death 1 ligand 1.

The anti-PD1/PDL1 antibodies evaluated in the 100 most cited papers are shown in **Table 5**. Most studies focused on nivolumab (42 papers, 71,606 citations) or pembrolizumab (34 papers, 43,352 citations). The research trend of immunotherapy drugs for different cancers is shown in **Figure 4B**. Early studies mainly evaluated nivolumab. The clinical trials of nivolumab or pembrolizumab investigated multiple cancers, while the frequently cited studies of atezolizumab and durvalumab mainly focused on lung cancer, urothelial cancer, or renal cell cancer.

**Table 5:** Types of drugs investigated in the top-cited papers on anti-PD1/PDL1 immunotherapy.

Drug types	Number of papers	Total number of citations	Average number of citations (per paper)		Average number of citations per year (per paper)
Nivolumab	42	71,606	1,704.9	295.4	
Pembrolizumab	34	43,352	1,275.1	273.6	
Atezolizumab	12	13,863	1,155.3	308.6	
Durvalumab	6	4,686	781	209.8	
Avelumab	3	1,968	656	177.5	
Pidilizumab	1	405	405	50.6	
Lambrolizumab	1	2,446	2,446	287.8	
Cemiplimab	1	514	514	146.9	

PD1, programmed cell death 1; PDL1, programmed cell death 1 ligand 1.

The relationship between cancers, anti-PD1/PDL1 antibodies, and research type is shown in **Figure 4C**. Most randomized phase 3 trials aimed to evaluate nivolumab or pembrolizumab for cancer immunotherapy. The indications of anti-PD1/PDL1 antibodies, approved by the US Food and Drug Administration (FDA) until January 1, 2022, are shown in **Figure 4D**. The FDA approved the use of nivolumab and pembrolizumab for immunotherapy in multiple cancers.

### Keywords and Research Hotspots

**Figure 5:**
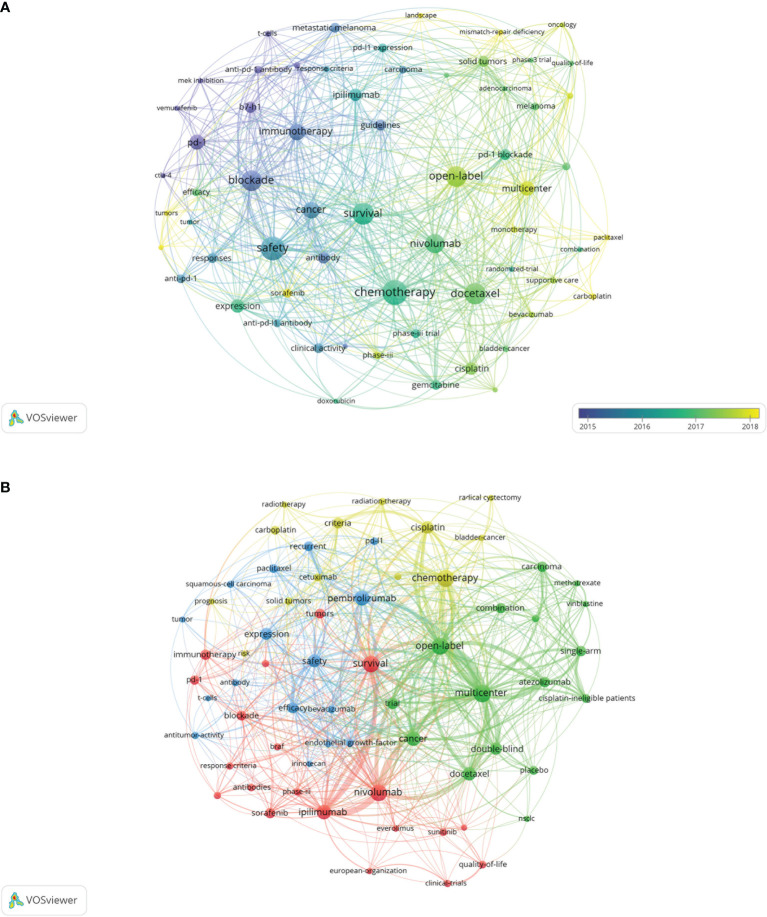
**(A)** Network visualization of keywords from the 100 most cited papers according to the average year of publication. **(B)** Network visualization of keywords from papers published in major journals from 2020 to 2021 according to cluster analysis. The circle size represents the number of articles included in the 100 most cited articles; the width of the curved line represents the link strength; and the distance between two keywords approximates the relatedness of the nodes. NSCLC, non-small cell lung cancer.

The keyword co-occurrence network of the 100 most frequently cited papers is shown in **Figure 5A**. The top keywords included “nivolumab”, “chemotherapy”, “safety”, “docetaxel”, “pneumonitis”, “open-label”, and “multicenter”. Recently utilized keywords included “mismatch-repair deficiency”, “sorafenib”, “monotherapy”, and “carboplatin”. The keyword co-occurrence network of 188 papers published on major journals after 2020 is shown in **Figure 5B**. The newly utilized keywords included “cetuximab”, “radiotherapy”, “methotrexate”, “vinblastine”, “BRAF”, “everolimus”, and “irinotecan”.

## Discussion

In recent years, anti-PD1/PDL1 immunotherapy has been used for the treatment of multiple types of cancer. The preliminary clinical trials confirmed the efficacy and safety of anti-PD1/PDL1 antibodies against refractory advanced cancers (9, 23, 24). Subsequent trials evaluated the usefulness of anti-PD1/PDL1 therapy for different types of cancer and stages of disease, and revealed surprising efficacy in select patients (13, 16, 25, 26). Based on these results, several anti-PD1 antibodies (e.g., pembrolizumab, nivolumab, and cemiplimab) and anti-PDL1 antibodies (e.g., atezolizumab, avelumab, and durvalumab) have been approved for certain indications by the US FDA. In recent years, anti-PD1/PDL1 therapy has become the standard treatment for various types of cancer. Further studies are underway to explore potential biomarkers, patient selection, specific therapeutic strategies, and the management of immune-related adverse effects.

### Biomarkers of Anti-PD1/PDL1 Immunotherapy

Although anti-PD1/PDL1 immunotherapy has improved patient outcomes in a variety of tumors, only a minority of patients treated with anti-PD1/PDL1 antibodies achieve a durable response (27). Some biomarkers relevant to response after anti-PD1/PDL1 therapy have been used to select patients and predict prognosis.

The expression levels of PDL1, which is positively correlated with response to treatment with ICIs in several cancer microenvironments, is one of the most commonly used response-predicting biomarkers of anti-PD1/PDL1 therapy (28–30). The expression of PDL1 by the tumor ensures the existence of targetable PD1:PDL1 interactions in the tumor microenvironment, and correlates with potential immune activation in the tumor (29). Thus, tumors with no or low PDL1 expression are likely to be resistant to anti-PD1/PDL1 therapy. Although this inference is supported by several trials, PDL1-negative tumors occasionally respond to anti-PD1/PDL1 therapy (28, 30). Moreover, overexpression of PDL1 in non-small cell lung cancer (NSCLC) may reduce the efficacy of anti-PD1 therapy. Notably, melanoma patients with moderate expression of PDL1 exhibited the best response to anti-PD1 therapy (31, 32).

High tumor mutation burden (TMB-H), defective DNA mismatch repair (dMMR), and high microsatellite instability (MSI-H) are strongly associated with response to anti-PD1/PDL1 therapy in multiple types of cancer (27). These genetic abnormalities lead to high neoantigen load, thus facilitating the recognition of the tumor by the immune system and resulting in a more potent anti-tumor response following treatment with an ICI (27). Clinical trials have confirmed the predictive value of these biomarkers in various tumors (33–35). However, a minority (~5%) of patients with low TMB respond well to ICIs, whereas >50% of patients with TMB-H do not respond at all (36).

Interferon-gamma (IFNG), which is an important cytokine for initiating and maintaining a potent anti-tumor response in multiple pathways, has been considered a useful predictor of response to anti-PD1/PDL1 therapy across various tumors (29, 37). IFNG is important for anti-tumor immune activity; research has shown that the tumor genetic mutations that disrupt the IFNG pathway signaling facilitate resistance to ICIs. For example, tumors with mutations in genes, such as Janus kinase 1/2 (JAK1/2) and interferon-gamma receptor 1/2 (IFNGR1/2), may result in resistance to ICI therapy (38, 39).

In addition, other biomarkers (e.g., T cell inflamed gene expressed profile, tumor infiltration lymphocytes, microbiome, and tumor burden) are valuable predictors of response to ICIs (40, 41). Considering the complexity of the immune system, multiple biomarkers should be incorporated into a composite system to ensure more accurate outcome prediction and patient selection, as well as fully unlock the potential benefit of immunotherapy. Moreover, individualized, in-depth, immune profiling is necessary to tailor treatment strategies on a patient-by-patient basis (36). Randomized trials are required to establish the roles of these biomarkers in various clinical settings.

### Anti-PD1/PDL1 in NSCLC

Of the 100 most cited papers, 27 focused on NSCLC. In 2015, two phase 3 randomized trials (CheckMate 017/057) reported significantly greater efficacy and safety of nivolumab versus docetaxel as second-line treatment for either squamous or non-squamous advanced NSCLC; of note, this efficacy was independent of the PDL1 expression (13, 15). In 2016, two randomized trials reported that both pembrolizumab (KEYNOTE-010) and atezolizumab (POPLAR) were superior to docetaxel as second-line treatment for NSCLC (42, 43). The KEYNOTE-010 trial only included patients with PDL1 expression, and suggested that patients with a PDL1 tumor proportion score (TPS) ≥50% had a better prognosis after treatment with pembrolizumab (42). Similarly, the POPLAR trial reported that PDL1 expression on tumor cells and tumor-infiltrating lymphocytes was correlated with better efficacy (43). Anti-PD1/PDL1 monotherapy has become the standard second-line treatment for NSCLC, and a portion of patients achieve long-term survival; nevertheless, the objective response rate (ORR) remains unsatisfactory. In a recent phase 3 randomized trial, the addition of ipilimumab to nivolumab did not improve outcomes in patients with pretreated advanced squamous NSCLC (44).

After 2016, the top-cited trials mostly aimed to evaluate anti-PD1/PDL1 therapy in the first-line setting for NSCLC. According to the KEYNOTE-024 phase 3 randomized trial, first-line pembrolizumab monotherapy showed better efficacy and safety than platinum-based chemotherapy in patients with advanced NSCLC and PDL1 TPS ≥50% (14). Although patients in the chemotherapy group with the progressive disease could switch treatment to pembrolizumab, the updated analysis was consistent with the initial result (45). The CheckMate 026 phase 3 randomized trial evaluated nivolumab as first-line treatment compared with platinum-based chemotherapy for patients with metastatic or recurrent NSCLC and PDL1 TPS ≥5%. Although superior safety was observed in patients receiving nivolumab, the overall survival (OS) and progression-free survival (PFS) were non-superior to those noted for chemotherapy (46). In contrast, the KEYNOTE-042 phase 3 randomized trial demonstrated that first-line pembrolizumab monotherapy results in superior efficacy and safety versus chemotherapy in patients with advanced NSCLC and PDL1 TPS ≥1%. Even patients with PDL1 TPS 1–20% who received pembrolizumab had a better prognosis than those treated with chemotherapy (47). The IMpower110 phase 3 randomized trial reported similar results in patients with advanced NSCLC and PDL1 expression on ≥1% of tumor cells or tumor-infiltrating lymphocytes who received first-line atezolizumab monotherapy (48). Regarding first-line combination therapy for advanced NSCLC, the addition of pembrolizumab or atezolizumab to chemotherapy significantly prolonged OS and PFS versus chemotherapy alone (49, 50). Additionally, several trials evaluated nivolumab plus anti-CTLA4 antibodies with or without chemotherapy as first-line treatment for NSCLC, and suggested that nivolumab plus anti-CTLA4 antibodies may be a promising modality (33, 51–53). However, the KEYNOTE-598 phase 3 randomized trial reported that the addition of ipilimumab to pembrolizumab showed greater toxicity without improvement in efficacy in previously untreated patients with metastatic NSCLC and PDL1 TPS ≥50% (54). Furthermore, T cell immunoreceptor with Ig and ITIM domains (TIGIT), a novel co-inhibitory receptor of T cell and natural killer cell activity, has become a recent popular target in cancer immunotherapy. In December 2021, the CITYSCAPE phase 2 randomized trial revealed that the addition of the anti-TIGIT antibody tiragolumab to atezolizumab significantly improved the efficacy and was associated with similar tolerability versus those observed with atezolizumab plus placebo as first-line treatment for metastatic NSCLC. The clinical benefit was more pronounced in the subgroup with PDL1 TPS ≥50% (55).

Given the excellent efficacy of anti-PD1/PDL1 against advanced NSCLC, researchers further evaluated the effectiveness of anti-PD1/PDL1 antibodies as adjuvant treatment for stages I–III NSCLC. In the PACIFIC phase 3 randomized trial, durvalumab significantly prolonged OS and PFS versus placebo in patients with stage III NSCLC after chemoradiotherapy (56). Data from a recent phase 2 trial (KEYNOTE-799) suggested promising antitumor activity of pembrolizumab plus chemoradiotherapy and manageable safety in patients with stage III NSCLC (57). A phase 2 single-arm trial evaluated treatment with neoadjuvant atezolizumab and chemotherapy in patients with resectable NSCLC; a high proportion (57%) of patients achieved major pathological response (58). Another phase 2 trial demonstrated that neoadjuvant nivolumab plus chemotherapy for resectable stage IIIA NSCLC results in a 2-year PFS rate of 77.1% (59). Moreover, a recent phase 2 randomized trial reported that the combination of neoadjuvant durvalumab with stereotactic body radiotherapy was associated with a significantly higher major pathological response rate versus durvalumab monotherapy (53.3% vs. 6.7%, respectively) in early-stage NSCLC (60). A recent phase 3 randomized trial (IMpower 010) showed a disease-free survival benefit with atezolizumab versus best supportive care after adjuvant chemotherapy in patients with resected stages II–IIIA NSCLC; the benefit was more pronounced in the subgroup with PDL1 TPS ≥1% (61). The combination of stereotactic ablative radiotherapy (SABR) with immunotherapy (I-SABR) for NSCLC is a research hotspot (11). The PEMBRO-RT phase 2 randomized trial demonstrated that SABR prior to pembrolizumab for locally advanced NSCLC improved the prognosis of patients. Interestingly, positive results were largely observed in the PDL1-negative subgroup (62). Recently, a phase 2 randomized trial demonstrated that neoadjuvant durvalumab plus SABR for early-stage NSCLC was well tolerated and associated with a high pathologic response rate (60).

Anti-PD1/PDL1 therapy is most widely studied in NSCLC compared with other cancers. Based on its encouraging efficacy, anti-PD1/PDL1 has become the standard treatment for advanced NSCLC without driver gene mutation. Some trials have demonstrated that adjuvant anti-PD1/PDL1 therapy improved the prognosis of patients with stages I–III NSCLC. However, the currently available clinical evidence is insufficient. Notably, I-SABR showed a significant synergistic effect. However, further studies are warranted to investigate the molecular mechanisms underlying the effects of I-SABR, and the influence of SABR on the tumor microenvironment.

### Anti-PD1/PDL1 in Melanoma

Prior to 2010, advanced melanoma was uniformly considered fatal with an OS <5% (63). Over the past decade, the advent of immunotherapy and molecular targeted therapy has revolutionized the treatment of advanced melanoma (64). A few years after the approval of ipilimumab for the treatment of advanced melanoma, the CheckMate 067 and KEYNOTE-006 phase 3 randomized trials showed significantly better efficacy and tolerability for nivolumab and pembrolizumab versus ipilimumab (8, 17). In the CheckMate 067 trial, the 5-year OS rates linked to the nivolumab-plus-ipilimumab, nivolumab, and ipilimumab groups were 52%, 44%, and 26%, respectively. In addition, dual treatment with ICIs also improved PFS and ORR compared single agent therapy; however, dual treatment was associated with a higher incidence of grade 3/4 toxicity (65). Despite the occurrence of severe toxicities resulting in treatment interruption or discontinuation, the prognosis of patients who discontinued treatment because of immune-related adverse events was similar to that observed for the overall population (65). The findings of the CheckMate 204 phase 2 randomized trial suggested that dual ICI therapy was superior to single-agent anti-PD1 therapy in melanoma patients with asymptomatic brain metastases (66). Predictive models incorporating clinical, genomic, and transcriptomic features have been developed to predict intrinsic resistance to anti-PD1 therapy; this combination appears to be more robust than the individual features (67). However, additional clinical evidence is required to determine the subset of patients for whom dual ICI therapy should be preferred.

Targeted therapy has shown high response rates, while immunotherapy has been linked to durable responses. Hence, the combination of these modalities for the treatment of patients with B-Raf proto-oncogene, serine/threonine kinase (BRAF)-mutant melanoma is worthy of investigation (64). In the KEYNOTE-022 phase 2 randomized trial, the combination of pembrolizumab with BRAF/MEK targeted therapy for the treatment of patients with BRAF(V600)-mutant melanoma led to substantial improvement in prognosis; however, it was also linked to a higher incidence of toxicity (68). In the IMspire150 phase 3 randomized trial, addition of atezolizumab to BRAF/MEK targeted therapy in patients with BRAF(V600)-mutation advanced melanoma was tolerable and significantly increased PFS (69). Furthermore, a recent phase 1/2 trial (PIVOT-02) reported an extended PFS and an acceptable rate of grade 3/4 adverse effects in response to the administration of bempegaldesleukin (a CD122-preferential interleukin 2 pathway agonist) plus nivolumab as first-line treatment for metastatic melanoma (70).

High-risk patients with resected melanoma require adjuvant therapy to reduce the risk of recurrence. In 2017, only 2 years after the approval of ipilimumab for the adjuvant treatment of patients with stage III melanoma, the CheckMate-238 phase 3 randomized trial reported that adjuvant therapy with nivolumab resulted in better efficacy and tolerability than adjuvant therapy with ipilimumab among patients with resected advanced melanoma (71). The KEYNOTE-054 phase 3 randomized trial of adjuvant therapy with pembrolizumab yielded similar results (72). The recent IMMUNED phase 2 randomized trial further evaluated adjuvant nivolumab plus ipilimumab versus nivolumab monotherapy in patients with completely resected stage IV melanoma. Adjuvant therapy with dual ICIs resulted in improved 2-year recurrence-free survival rate (70% vs. 42%, respectively) and higher incidence of treatment-related grades 3–4 adverse events (71% vs. 27%, respectively) compared with nivolumab monotherapy (73). However, given the comparable improvement in recurrence-free survival, the selection of therapeutic option for patients eligible for both adjuvant anti-PD1 therapy and BRAF/MEK targeted therapy is a challenge (64).

Advances in ICIs and targeted therapy have markedly improved the prognosis of patients with advanced melanoma (64). Although dual ICI therapy resulted in greater efficacy than anti-PD1 monotherapy or targeted therapy, its clinical application is limited by the high incidence of immune-related adverse events. Combination of ICIs and targeted therapy appears to further improve the prognosis of advanced melanoma; nevertheless, additional high-level clinical evidence is needed. Future studies are warranted to overcome resistance, identify novel biomarker strategies, and accelerate precision medicine.

### Anti-PD1/PDL1 in Other Cancers

Anti-PD1/PDL1 antibodies have been used for the treatment of various cancers (e.g., lymphoma). Given the high response and recurrence rates recorded after systemic therapy for lymphoma, immunotherapy was usually utilized against relapsed or refractory lymphoma. Thus, despite the lack of evidence from randomized trials, the US FDA approved the use of anti-PD1 antibodies as salvage treatment for lymphoma (74). The recent KEYNOTE-204 phase 3 randomized trial reported improved efficacy with pembrolizumab versus brentuximab vedotin for refractory classical Hodgkin lymphoma (75). Moreover, the NIVAHL phase 2 randomized trial revealed excellent 1-year PFS and an unexpectedly high complete response rate after concomitant or sequential administration of nivolumab and doxorubicin, vinblastine, and dacarbazine as first-line treatment for early-stage unfavorable classic Hodgkin lymphoma (76).

In 2015, the CheckMate 025 phase 3 randomized trial demonstrated better efficacy and tolerability with nivolumab versus everolimus in previously treated patients with renal cell cancer (25). Subsequent trials further confirmed the superiority of several anti-PD1/PDL1 antibodies as part of a combination therapy for the first-line treatment of advanced renal cell cancer (77–80). Recently, the results of the KEYNOTE-564 phase 3 randomized trial supported the use of pembrolizumab as an adjuvant therapy following the resection of renal cell cancer (81).

Platinum-based chemotherapy is an established standard care for advanced urothelial cancer. In recent years, anti-PD1/PDL1 therapy became a standard treatment for platinum-refractory or platinum-ineligible patients with advanced urothelial cancer (82–84). Several trials evaluated anti-PD1/PDL1 therapy as first-line treatment for urothelial cancer. However, except for the addition of atezolizumab to chemotherapy, other attempts failed to offer benefit (85–87). Adjuvant therapy for urothelial cancer is a new research hotspot. The IMvigor010 phase 3 randomized trial of adjuvant atezolizumab for resected high-risk muscle-invasive urothelial cancer reported negative results (88). However, the recent CheckMate 274 phase 3, randomized, placebo-controlled trial suggested that adjuvant nivolumab prolonged the disease-free survival of patients with resected high-risk muscle-invasive urothelial cancer and PDL1 TPS ≥1% (89). Furthermore, the use of avelumab or pembrolizumab as neoadjuvant therapy in patients with non-metastatic muscle invasive bladder cancer is investigated is ongoing trials (Oncodistinct 004-AURA, KEYNOTE-866, KEYNOTE-905) (90, 91).

Initially, anti-PD1/PDL1 therapy for gastric or gastro-esophageal junction cancer (GC) was used as salvage treatment. The ATTRACTION-2 phase 3 randomized trial demonstrated improved prognosis with nivolumab versus placebo for heavily pretreated patients with advanced GC (92). However, in the KEYNOTE-061 phase 3 randomized trial, although pembrolizumab resulted in better safety, it did not significantly improve OS compared with paclitaxel as second-line therapy for patients with advanced GC and PDL1 combined positive score ≥1 (93). Similarly, in the KEYNOTE-062 trial, pembrolizumab alone or in combination with chemotherapy was not superior to chemotherapy for untreated patients with advanced GC (94). Encouragingly, the recent KEYNOTE-811 phase 3 randomized trial reported that addition of pembrolizumab to trastuzumab and chemotherapy resulted in a substantial, statistically significant increase in ORR versus trastuzumab and chemotherapy alone for untreated patients with human epidermal growth factor receptor 2 (HER2)-positive metastatic GC; responses were durable and safety was manageable (95). Currently, there is limited high-level clinical evidence supporting the use of anti-PD1/PDL1 as first- or second-line treatment for HER2-negative advanced GC.

A proportion of colorectal cancers have the genetic alteration MSI-H/dMMR, which indicates potential response after immunotherapy. Preliminary data of the CheckMate 142 phase 2 trial suggested that nivolumab with/without ipilimumab led to durable responses and disease control in pretreated patients with MSI-H/dMMR metastatic colorectal cancer (96). The KEYNOTE-177 phase 3 randomized trial demonstrated that pembrolizumab was superior to chemotherapy with respect to PFS for untreated patients with advanced colorectal cancer with MSI-H/dMMR (97). However, microsatellite-stable colorectal cancer is typically unresponsive to immunotherapy. The IMblaze370 phase 3 randomized trial of atezolizumab plus cobimetinib or atezolizumab monotherapy versus regorafenib as third-line treatment for metastatic colorectal cancer with microsatellite-stable reported negative results (98).

The treatment of small cell lung cancer (SCLC) has gradually advanced in the past two decades. Phase 3 randomized trials demonstrated that the addition of atezolizumab, durvalumab, or pembrolizumab to chemotherapy improved the prognosis of untreated patients with extensive-stage SCLC (99–101). Anti-PD1 plus concurrent chemoradiotherapy (CCRT) for limited-stage SCLC is a current research hotspot. The STIMULI phase 2 randomized trial did not meet its primary endpoint of improving PFS with nivolumab-ipilimumab consolidation after CCRT in limited-stage SCLC (102). Furthermore, the KEYLYNK-013 trial evaluating the addition of pembrolizumab to CCRT for limited-stage SCLC is ongoing.

Several trials contributed to the approval of anti-PD1/PDL1 as first-/second-line treatment for various types of advanced cancers (e.g., hepatocellular cancer, head and neck squamous cell cancer, and triple-negative breast cancer) (22, 26, 103–105). The KEYNOTE-522 phase 3 randomized trial suggested that adding pembrolizumab to platinum-based neoadjuvant chemotherapy significantly increased the percentage of patients with a pathological complete response among those with locally advanced triple-negative breast cancer (106).

### Journals, Countries, Institutions, and Authors

*N. Engl. J. Med.* has published 40 of the 100 most cited papers on clinical studies of anti-PD1/PDL1 immunotherapy. The number of top-cited papers, average citations per paper, and rate of top-cited papers of *N. Engl. J. Med.* is markedly higher than that of other journals. Thus, *N. Engl. J. Med.* is undoubtedly the most influential journal regarding clinical studies of anti-PD1/PDL1 immunotherapy. *Lancet* has the second highest average number of citations per paper and rate of top-cited papers among the journals, indicating that it is a highly influential journal. *Lancet Oncology*, *Journal of Clinical Oncology*, *Journal of the American Medical Association* (*JAMA*) *Oncology*, and *JAMA* have published a portion of the top-cited papers, and are considered major journals regarding clinical studies of anti-PD1/PDL1 immunotherapy.

Although most corresponding authors of the top-cited papers were based in the USA, authors from 40 countries or regions contributed to these studies. International and interagency cooperation was common because most of these studies were initiated by large multinational pharmacy enterprises. Recently published large trials included patients from developing countries. However, there is a lack of data for patients from Africa. Renowned institutions from the USA have contributed greatly to these trials. The most prolific authors were Brahmer JR, Hamid O, and Hodi FS, while Hodi FS, Motzer RJ, and Topalian SL had the highest corresponding author frequency.

### Research Areas

Anti-PD1/PDL1 immunotherapy mainly targets NSCLC and melanoma. Given its high morbidity and demonstrated benefit from anti-PD1/PDL1 therapy, NSCLC attracts considerable attention in this area. Anti-PD1/PDL1 therapy has become a standard treatment for advanced NSCLC without driver gene mutation. Additionally, ongoing studies evaluate the use of anti-PD1/PDL1 in more indications. Although the morbidity of melanoma is markedly lower than that of NSCLC, anti-PD1/PDL1 therapy has revolutionized its treatment based on the favorable efficacy. Moreover, investigations of anti-PD1/PDL1 therapy for other cancers have gradually changed the treatment paradigm.

Thus far, most top-cited papers focused on nivolumab and pembrolizumab, as these two anti-PD1 antibodies have been approved for several indications in cancer immunotherapy. Atezolizumab is the most studied anti-PDL1 antibody; it is thought that atezolizumab is more tolerable than anti-PD1 antibodies, and has been approved mainly for the treatment of advanced urothelial cancer, lung cancer, melanoma, and hepatocellular cancer. Durvalumab is the second most studied anti-PDL1 antibody, and the only immunotherapy agent approved as adjuvant treatment following chemoradiotherapy for stage-III NSCLC.

### Research Trends, Current Status, and Hotspots

The analysis of the popular keywords in the top-cited papers and newest papers from major journals revealed the research trends and hotspots of anti-PD1/PDL1 immunotherapy. The initial studies mainly investigated the clinical efficacy and safety of anti-PD1/PDL1 therapy for non-specific cancer, and subsequently compared anti-PD1/PDL1 therapy with anti-CTLA4 antibodies and targeted therapies for melanoma. Once the superiority of anti-PD1/PDL1 therapy was confirmed, further studies evaluated the use of anti-PD1/PDL1 therapy for various cancers. Studies demonstrated that anti-PD1/PDL1 therapy was superior to paclitaxel or platinum-based chemotherapy in some cancers, and established anti-PD1/PDL1 therapy as a standard treatment for multiple types of advanced cancers. Also, the research hotspots continued to change. The most recent papers from major journals mainly focused on the combination of anti-PD1/PDL1 therapy and other treatments, management of immune-related adverse events, patient selection, and adjuvant therapy.

Based on the results of the bibliometric analysis and recent important advances, the present study summarized the current research status and hotspots of anti-PD1/PDL1 immunotherapy. Firstly, the value and indications of anti-PD1/PDL1 immunotherapy for common advanced cancers have been clarified. Anti-PD1/PDL1 immunotherapy has become an important standard treatment for various cancers. Secondly, some biomarkers (e.g., PDL1 expression, TMB, and MSI/MMR) are valuable in patient selection. More comprehensive and robust predictive biomarkers are required to further determine the patients who can potentially benefit from anti-PD1/PDL1 immunotherapy. Thirdly, the combination of anti-PD1/PDL1 and anti-CTLA4, chemotherapy, targeted therapy, or anti-vascular therapy may further improve the prognosis. Further investigation is warranted to determine the optimal therapeutic strategies for advanced cancers. Fourthly, I-SABR for various cancers is an interesting option. Further studies are ongoing to clarify the clinical benefit and molecular mechanism involved in this treatment. Fifthly, the addition of anti-PD1/PDL1 antibodies to radical treatment has improved the prognosis of non-metastatic NSCLC, melanoma, renal cell cancer, bladder cancer, and triple-negative breast cancer. Neoadjuvant and adjuvant immunotherapy, and the combination of immunotherapy with CCRT are current research hotspots.

### Limitations

This study had certain limitations. Firstly, the number of citations of papers was influenced by various confounding factors (e.g., time of publication, research area, journal, and author). Therefore, this number may not accurately represent the influence of a paper. Most of the top-cited papers analyzed in this study were published prior to 2021; hence, some recent important publications may have been omitted, and the top-cited papers may not represent the latest research hotspots. To minimize the impact of publication time, we analyzed the average number of citations per year of the papers. To determine the current research hotspots, we identified papers published in major journals within the last 2 years (2020–2021), searched for high-impact papers, and analyzed their findings. Secondly, half of the selected papers focused on lung cancer or melanoma; nevertheless, other less common cancers are also important. Because different subdomains of research were grouped together in a single bibliometric analysis, smaller research areas were not represented. To address this problem, we discussed important advances in each subdomain. Thirdly, although the document labels of the Web of Science are more accurate than other databases with citation information such as Scopus, the labels still might be wrong (21). To overcome this, we carefully read the papers and correctly classified the articles. Fourthly, we identified articles only using the Web of Science search engine; thus, publications in other databases or articles that were written in a language other than English may have been missed. This may have introduced bias into the analysis of citations and resulted in the omission of important evidence. Finally, although we determined the research area and study design for each paper, a more detailed analysis of these articles was not possible; consequently, non-major findings were not noted.

## Conclusion

The present bibliometric analysis provides insight into the historical development and important advances in anti-PD1/PDL1 therapy, and highlights the current research hotspots. Anti-PD1/PDL1 immunotherapy has become the standard treatment for various cancers, while *N. Engl. J. Med.* is undoubtedly the most influential journal in this area. The current research status is as follows: (1) Immunotherapy for late-stage cancers has been preliminary studied, but clinical evidence for adjuvant/neo-adjuvant immunotherapy is insufficient. (2) Lots of clinical trials have evaluated the efficacy and safety of single agent immunotherapy or combination of immunotherapy with chemotherapy or antiangiogenic therapy. However, the combination of immunotherapy with radiotherapy or targeted therapy is less investigated. (3) NSCLC and melanoma have been heavily studied, but the approval of new indications of immunotherapy in other cancers needs further studies. (4) Some biomarkers have been established to predict the efficacy of immunotherapy. Nevertheless, their sensitivity and specificity still need to be improved. Further studies are warranted to identify superior predictive biomarkers or models, clarify the molecular mechanism involved in combination therapy, and establish optimal therapeutic strategies. This study may assist researchers in obtaining a comprehensive impression of the landscape and current trends in anti-PD1/PDL1 immunotherapy and gain inspiration to conduct further studies.

## Data Availability Statement

The raw data supporting the conclusions of this article will be made available by the authors, without undue reservation.

## Author Contributions

XZ and YHL contributed to the study conception. YHL analyzed the data. YHL, YX, SJ, YL, HY, ZZ, and LL contributed to the literature review. YHL wrote the manuscript. All authors contributed to the article and approved the submitted version.

## Conflict of Interest

The authors declare that the research was conducted in the absence of any commercial or financial relationships that could be construed as a potential conflict of interest.

## Publisher’s Note

All claims expressed in this article are solely those of the authors and do not necessarily represent those of their affiliated organizations, or those of the publisher, the editors and the reviewers. Any product that may be evaluated in this article, or claim that may be made by its manufacturer, is not guaranteed or endorsed by the publisher.

## Supplementary Material

Supplementary Material S1 Detailed search strategy for the 100 most cited clinical studies on anti-PD1/PDL1 immunotherapy. PD1, programmed cell death 1; PDL1, programmed cell death 1 ligand 1.

Supplementary Table S1 The 100 most cited clinical studies on anti-PD1/PDL1 immunotherapy. PD1, programmed cell death 1; PDL1, programmed cell death 1 ligand 1.

Supplementary Figure 1 Historical direct citation network among the most cited papers.
