# Supplementary material for: Research Trends and Most Influential Clinical Studies on Anti-PD1/PDL1 Immunotherapy for Cancers: A Bibliometric Analysis
Source: Front Immunol. 2022 Apr 11;13:862084. doi: 10.3389/fimmu.2022.862084 (PMC9044908; doi:10.3389/fimmu.2022.862084)
Supplement: Supplementary Material S1 — Detailed search strategy for the 100 most cited clinical studies on anti-PD1/PDL1 immunotherapy. PD1, programmed cell death 1; PDL1, programmed cell death 1 ligand 1. [file DataSheet_1.docx]

(TS= ((cancer) OR (carcinoma) OR (neoplasm) OR (adenocarcinoma) OR (melanoma) OR (adenocarcinoma) OR (sarcoma) OR (osteosarcoma)) AND TS=((checkpoint inhibitor) OR (checkpoint inhibitors) OR (checkpoint blockade) OR (PD1) OR (PD-1) OR (PD-L1) OR (anti-PD1) OR (anti-PD-1) OR (anti-PD-L1) OR (PD1/PD-L1) OR (PD-1/PD-L1) OR (anti-PD1/PD-L1) OR (anti-PD-1/PD-L1) OR (atezolizumab) OR (avelumab) OR (durvalumab) OR (nivolumab) OR (pembrolizumab) OR (tislelizumab) OR (camrelizumab) OR (penpulimab) OR(toripalimab) OR (sintilimab) OR (lambrolizumab) OR (pidilizumab) OR (cemiplimab))) AND DT=(Article) NOT TI=((guideline) OR (consensus recommendations) OR (meta-analyses) OR (meta-analysis) OR (meta analysis) OR (data pooling) OR (pooled) OR (overview) OR (mouse) OR (mice) OR (animal) OR (rat) OR (rats) OR (in vitro) OR (invitro) OR (current status) OR (development) OR (review) OR (CTLA-4) OR (BRAF-mutated) OR (T-Cell Transfer) OR (management) OR (fulminant myocarditis) OR (effect in a patient) OR (immunohistochemistry assays) OR (prognostic and predictive value) OR (quantitative assessment) OR (RECIST) OR (a pan-cancer analysis) OR (Hu5F9-G4) OR (tumour-infiltrating CD8 cells))
