# Supplementary material for: Research Trends and Most Influential Clinical Studies on Anti-PD1/PDL1 Immunotherapy for Cancers: A Bibliometric Analysis
Source: Front Immunol. 2022 Apr 11;13:862084. doi: 10.3389/fimmu.2022.862084 (PMC9044908; doi:10.3389/fimmu.2022.862084)
Supplement: Supplementary Table S1 — The 100 most cited clinical studies on anti-PD1/PDL1 immunotherapy. PD1, programmed cell death 1; PDL1, programmed cell death 1 ligand 1. [file Table_1.docx]

|  | **TABLE S1** The 100 most cited clinical studies in anti-PD1/PDL1 immunotherapy. | | | | | | | |
| --- | --- | --- | --- | --- | --- | --- | --- | --- |
| Rank | | Title | corresponding author | Journal | Year | Total citation | Average citation per year (rank) |  |
| 1 | | Safety, Activity, and Immune Correlates of Anti-PD-1 Antibody in Cancer | Topalian SL | N. Engl. J. Med. | 2012 | 7983 | 833.01 (3) |  |
| 2 | | Nivolumab versus Docetaxel in Advanced Nonsquamous Non-Small-Cell Lung Cancer | Borghaei H | N. Engl. J. Med. | 2015 | 5521 | 883.36 (2) |  |
| 3 | | Safety and Activity of Anti-PD-L1 Antibody in Patients with Advanced Cancer | Brahmer JR | N. Engl. J. Med. | 2012 | 5052 | 527.17 (11) |  |
| 4 | | PD-1 Blockade in Tumors with Mismatch-Repair Deficiency | Diaz LA | N. Engl. J. Med. | 2015 | 4938 | 750.08 (4) |  |
| 5 | | Pembrolizumab versus Chemotherapy for PD-L1-Positive Non-Small-Cell Lung Cancer | Brahmer JR | N. Engl. J. Med. | 2016 | 4866 | 941.81 (1) |  |
| 6 | | Combined Nivolumab and Ipilimumab or Monotherapy in Untreated Melanoma | Hodi FS | N. Engl. J. Med. | 2015 | 4791 | 737.08 (5) |  |
| 7 | | Nivolumab versus Docetaxel in Advanced Squamous-Cell Non-Small-Cell Lung Cancer | Brahmer J | N. Engl. J. Med. | 2015 | 4405 | 677.69 (6) |  |
| 8 | | Pembrolizumab for the Treatment of Non-Small-Cell Lung Cancer | Garon EB | N. Engl. J. Med. | 2015 | 3618 | 542.7 (10) |  |
| 9 | | Nivolumab versus Everolimus in Advanced Renal-Cell Carcinoma | Motzer RJ | N. Engl. J. Med. | 2015 | 3453 | 559.95 (9) |  |
| 10 | | Nivolumab in Previously Untreated Melanoma without BRAF Mutation | Robert C | N. Engl. J. Med. | 2015 | 3444 | 492 (14) |  |
| 11 | | Pembrolizumab versus Ipilimumab in Advanced Melanoma | Robert C | N. Engl. J. Med. | 2015 | 3395 | 515.7 (13) |  |
| 12 | | Pembrolizumab versus docetaxel for previously treated, PD-L1-positive, advanced non-small-cell lung cancer (KEYNOTE-010): a randomised controlled trial | Herbst RS | Lancet | 2016 | 3028 | 526.61 (12) |  |
| 13 | | Nivolumab plus Ipilimumab in Advanced Melanoma | Wolchok JD | N. Engl. J. Med. | 2013 | 2892 | 340.24 (30) |  |
| 14 | | Pembrolizumab plus Chemotherapy in Metastatic Non-Small-Cell Lung Cancer | Gandhi L | N. Engl. J. Med. | 2018 | 2466 | 672.55 (7) |  |
| 15 | | Atezolizumab versus docetaxel in patients with previously treated non-small-cell lung cancer (OAK): a phase 3, open-label, multicentre randomised controlled trial | Gandara DR | Lancet | 2017 | 2447 | 489.4 (15) |  |
| 16 | | Safety and Tumor Responses with Lambrolizumab (Anti-PD-1) in Melanoma | Ribas A | N. Engl. J. Med. | 2013 | 2446 | 287.76 (37) |  |
| 17 | | PD-1 Blockade with Nivolumab in Relapsed or Refractory Hodgkin's Lymphoma | Ansell SM | N. Engl. J. Med. | 2015 | 2300 | 328.57 (33) |  |
| 18 | | Nivolumab for Recurrent Squamous-Cell Carcinoma of the Head and Neck | Gillison ML | N. Engl. J. Med. | 2016 | 2224 | 430.45 (18) |  |
| 19 | | Atezolizumab in patients with locally advanced and metastatic urothelial carcinoma who have progressed following treatment with platinum-based chemotherapy: a single-arm, multicentre, phase 2 trial | Rosenberg JE | Lancet | 2016 | 2116 | 373.41 (28) |  |
| 20 | | Phase I Study of Single-Agent Anti-Programmed Death-1 (MDX-1106) in Refractory Solid Tumors: Safety, Clinical Activity, Pharmacodynamics, and Immunologic Correlates | Topalian SL | J. Clin. Oncol. | 2010 | 1982 | 172.35 (61) |  |
| 21 | | Nivolumab in patients with advanced hepatocellular carcinoma (CheckMate 040): an open-label, non-comparative, phase 1/2 dose escalation and expansion trial | El-Khoueiry AB | Lancet | 2017 | 1833 | 399.93 (24) |  |
| 22 | | Nivolumab and Ipilimumab versus Ipilimumab in Untreated Melanoma | Hodi FS | N. Engl. J. Med. | 2015 | 1829 | 274.35 (38) |  |
| 23 | | Durvalumab after Chemoradiotherapy in Stage III Non-Small-Cell Lung Cancer | Antonia SJ | N. Engl. J. Med. | 2017 | 1789 | 429.36 (19) |  |
| 24 | | Nivolumab plus Ipilimumab versus Sunitinib in Advanced Renal-Cell Carcinoma | Motzer RJ | N. Engl. J. Med. | 2018 | 1776 | 473.6 (17) |  |
| 25 | | Overall Survival with Combined Nivolumab and Ipilimumab in Advanced Melanoma | Wolchok JD | N. Engl. J. Med. | 2017 | 1744 | 410.35 (23) |  |
| 26 | | Survival, Durable Tumor Remission, and Long-Term Safety in Patients With Advanced Melanoma Receiving Nivolumab | Topalian SL | J. Clin. Oncol. | 2014 | 1582 | 204.13 (51) |  |
| 27 | | Pembrolizumab as Second-Line Therapy for Advanced Urothelial Carcinoma | Bellmunt J | N. Engl. J. Med. | 2017 | 1568 | 324.41 (34) |  |
| 28 | | Atezolizumab and Nab-Paclitaxel in Advanced Triple-Negative Breast Cancer | Schmid P | N. Engl. J. Med. | 2018 | 1547 | 488.53 (16) |  |
| 29 | | Atezolizumab for First-Line Treatment of Metastatic Nonsquamous NSCLC | Reck M | N. Engl. J. Med. | 2018 | 1492 | 416.37 (21) |  |
| 30 | | Nivolumab plus Ipilimumab in Lung Cancer with a High Tumor Mutational Burden | Hellmann MD | N. Engl. J. Med. | 2018 | 1384 | 377.45 (27) |  |
| 31 | | Atezolizumab versus docetaxel for patients with previously treated non-small-cell lung cancer (POPLAR): a multicentre, open-label, phase 2 randomised controlled trial | Fehrenbacher L | Lancet | 2016 | 1368 | 237.91 (44) |  |
| 32 | | Pembrolizumab plus Chemotherapy for Squamous Non-Small-Cell Lung Cancer | Paz-Ares L | N. Engl. J. Med. | 2018 | 1347 | 425.37 (20) |  |
| 33 | | First-Line Nivolumab in Stage IV or Recurrent Non-Small-Cell Lung Cancer | Carbone DP | N. Engl. J. Med. | 2017 | 1346 | 293.67 (36) |  |
| 34 | | Anti-programmed-death-receptor-1 treatment with pembrolizumab in ipilimumab-refractory advanced melanoma: a randomised dose-comparison cohort of a phase 1 trial | Robert C | Lancet | 2014 | 1256 | 171.27 (62) |  |
| 35 | | Pembrolizumab in Patients With Advanced Triple-Negative Breast Cancer: Phase Ib KEYNOTE-012 Study | Nanda R | J. Clin. Oncol. | 2016 | 1134 | 206.18 (50) |  |
| 36 | | Overall Survival with Durvalumab after Chemoradiotherapy in Stage III NSCLC | Antonia SJ | N. Engl. J. Med. | 2018 | 1119 | 362.92 (29) |  |
| 37 | | Nivolumab in patients with metastatic DNA mismatch repair-deficient or microsatellite instability-high colorectal cancer (CheckMate 142): an open-label, multicentre, phase 2 study | Overman MJ | Lancet Oncol. | 2017 | 1103 | 254.54 (42) |  |
| 38 | | Pembrolizumab plus Axitinib versus Sunitinib for Advanced Renal-Cell Carcinoma | Rini BI | N. Engl. J. Med. | 2019 | 1097 | 387.18 (26) |  |
| 39 | | Adjuvant Nivolumab versus Ipilimumab in Resected Stage III or IV Melanoma | Weber J | N. Engl. J. Med. | 2017 | 1049 | 251.76 (43) |  |
| 40 | | Pembrolizumab versus chemotherapy for previously untreated, PD-L1-expressing, locally advanced or metastatic non-small-cell lung cancer (KEYNOTE-042): a randomised, open-label, controlled, phase 3 trial | Mok TSK | Lancet | 2019 | 1047 | 392.63 (25) |  |
| 41 | | Pembrolizumab versus investigator-choice chemotherapy for ipilimumab-refractory melanoma (KEYNOTE-002): a randomised, controlled, phase 2 trial | Ribas A | Lancet Oncol. | 2015 | 1033 | 160.99 (66) |  |
| 42 | | Atezolizumab plus Bevacizumab in Unresectable Hepatocellular Carcinoma | Cheng AL | N. Engl. J. Med. | 2020 | 1022 | 613.2 (8) |  |
| 43 | | First-Line Atezolizumab plus Chemotherapy in Extensive-Stage Small-Cell Lung Cancer | Horn L | N. Engl. J. Med. | 2018 | 1020 | 330.81 (32) |  |
| 44 | | Atezolizumab as first-line treatment in cisplatin-ineligible patients with locally advanced and metastatic urothelial carcinoma: a single-arm, multicentre, phase 2 trial | Balar AV | Lancet | 2017 | 1011 | 202.2 (52) |  |
| 45 | | Activity and safety of nivolumab, an anti-PD-1 immune checkpoint inhibitor, for patients with advanced, refractory squamous non-small-cell lung cancer (CheckMate 063): a phase 2, single-arm trial | Rizvi NA | Lancet Oncol. | 2015 | 989 | 144.73 (72) |  |
| 46 | | Nivolumab in patients with advanced gastric or gastro-oesophageal junction cancer refractory to, or intolerant of, at least two previous chemotherapy regimens (ONO-4538-12, ATTRACTION-2): a randomised, double-blind, placebo-controlled, phase 3 trial | Boku N | Lancet | 2017 | 937 | 229.47 (45) |  |
| 47 | | Five-Year Survival with Combined Nivolumab and Ipilimumab in Advanced Melanoma | Larkin J | N. Engl. J. Med. | 2019 | 931 | 413.78 (22) |  |
| 48 | | Carboplatin and pemetrexed with or without pembrolizumab for advanced, non-squamous non-small-cell lung cancer: a randomised, phase 2 cohort of the open-label KEYNOTE-021 study | Langer CJ | Lancet Oncol. | 2016 | 910 | 176.13 (58) |  |
| 49 | | Safety and clinical activity of pembrolizumab for treatment of recurrent or metastatic squamous cell carcinoma of the head and neck (KEYNOTE-012): an open-label, multicentre, phase 1b trial | Seiwert TY | Lancet Oncol. | 2016 | 907 | 164.91 (65) |  |
| 50 | | Pembrolizumab in patients with advanced hepatocellular carcinoma previously treated with sorafenib (KEYNOTE-224): a non-randomised, open-label phase 2 trial | Zhu AX | Lancet Oncol. | 2018 | 900 | 257.14 (40) |  |
| 51 | | Avelumab plus Axitinib versus Sunitinib for Advanced Renal-Cell Carcinoma | Motzer RJ | N. Engl. J. Med. | 2019 | 899 | 317.29 (35) |  |
| 52 | | Nivolumab in metastatic urothelial carcinoma after platinum therapy (CheckMate 275): a multicentre, single-arm, phase 2 trial | Sharma P | Lancet Oncol. | 2017 | 851 | 176.07 (59) |  |
| 53 | | Adjuvant Pembrolizumab versus Placebo in Resected Stage III Melanoma | Eggermont AMM | N. Engl. J. Med. | 2018 | 791 | 215.73 (46) |  |
| 54 | | Durable Clinical Benefit With Nivolumab Plus Ipilimumab in DNA Mismatch Repair-Deficient/Microsatellite Instability-High Metastatic Colorectal Cancer | Overman MJ | J. Clin. Oncol. | 2018 | 770 | 200.87 (53) |  |
| 55 | | Nivolumab alone and nivolumab plus ipilimumab in recurrent small-cell lung cancer (CheckMate 032): a multicentre, open-label, phase 1/2 trial | Calvo E | Lancet Oncol. | 2016 | 740 | 134.55 (75) |  |
| 56 | | Nivolumab plus Ipilimumab in Advanced Non-Small-Cell Lung Cancer | Hellmann MD | N. Engl. J. Med. | 2019 | 719 | 331.85 (31) |  |
| 57 | | PD-1 Blockade with Pembrolizumab in Advanced Merkel-Cell Carcinoma | Topalian SL | N. Engl. J. Med. | 2016 | 717 | 128.42 (76) |  |
| 58 | | Avelumab in patients with chemotherapy-refractory metastatic Merkel cell carcinoma: a multicentre, single-group, open-label, phase 2 trial | Kaufman HL | Lancet Oncol. | 2016 | 707 | 134.67 (74) |  |
| 59 | | Neoadjuvant PD-1 Blockade in Resectable Lung Cancer | Pardoll DM | N. Engl. J. Med. | 2018 | 706 | 192.55 (54) |  |
| 60 | | Nivolumab for Metastatic Renal Cell Carcinoma: Results of a Randomized Phase II Trial | Motzer RJ | J. Clin. Oncol. | 2015 | 698 | 104.7 (86) |  |
| 61 | | Safety and Efficacy of Pembrolizumab Monotherapy in Patients With Previously Treated Advanced Gastric and Gastroesophageal Junction Cancer Phase 2 Clinical KEYNOTE-059 Trial | Fuchs CS | JAMA Oncol. | 2018 | 658 | 179.45 (57) |  |
| 62 | | Atezolizumab versus chemotherapy in patients with platinum-treated locally advanced or metastatic urothelial carcinoma (IMvigor211): a multicentre, open-label, phase 3 randomised controlled trial | Powles T | Lancet | 2018 | 657 | 167.74 (63) |  |
| 63 | | Pembrolizumab for patients with PD-L1-positive advanced gastric cancer (KEYNOTE-012): a multicentre, open-label, phase 1b trial | Muro K | Lancet Oncol. | 2016 | 636 | 113.91 (77) |  |
| 64 | | Association of Pembrolizumab With Tumor Response and Survival Among Patients With Advanced Melanoma | Ribas A | JAMA-J. Am. Med. Assoc. | 2016 | 626 | 108.87 (81) |  |
| 65 | | Pembrolizumab versus ipilimumab for advanced melanoma: final overall survival results of a multicentre, randomised, open-label phase 3 study (KEYNOTE-006) | Schachter J | Lancet | 2017 | 618 | 145.41 (71) |  |
| 66 | | Safety and Antitumor Activity of Anti-PD-1 Antibody, Nivolumab, in Patients With Platinum-Resistant Ovarian Cancer | Hamanishi J | J. Clin. Oncol. | 2015 | 609 | 100.11 (89) |  |
| 67 | | Pembrolizumab for patients with melanoma or non-small-cell lung cancer and untreated brain metastases: early analysis of a non-randomised, open-label, phase 2 trial | Goldberg SB | Lancet Oncol. | 2016 | 598 | 108.73 (82) |  |
| 68 | | Nivolumab in Patients With Relapsed or Refractory Hematologic Malignancy: Preliminary Results of a Phase Ib Study | Lesokhin AM | J. Clin. Oncol. | 2016 | 596 | 110.03 (80) |  |
| 69 | | Pembrolizumab alone or with chemotherapy versus cetuximab with chemotherapy for recurrent or metastatic squamous cell carcinoma of the head and neck (KEYNOTE-048): a randomised, open-label, phase 3 study | Burtness B | Lancet | 2019 | 586 | 270.46 (39) |  |
| 70 | | Nivolumab plus ipilimumab or nivolumab alone versus ipilimumab alone in advanced melanoma (CheckMate 067): 4-year outcomes of a multicentre, randomised, phase 3 trial | Hodi FS | Lancet Oncol. | 2018 | 580 | 183.16 (55) |  |
| 71 | | First-line pembrolizumab in cisplatin-ineligible patients with locally advanced and unresectable or metastatic urothelial cancer (KEYNOTE-052): a multicentre, single-arm, phase 2 study | Balar AV | Lancet Oncol. | 2017 | 579 | 138.96 (73) |  |
| 72 | | Nivolumab for classical Hodgkin's lymphoma after failure of both autologous stem-cell transplantation and brentuximab vedotin: a multicentre, multicohort, single-arm phase 2 trial | Younes A | Lancet Oncol. | 2016 | 576 | 108 (84) |  |
| 73 | | Overall Survival and Long-Term Safety of Nivolumab (Anti-Programmed Death 1 Antibody, BMS-936558, ONO-4538) in Patients With Previously Treated Advanced Non-Small-Cell Lung Cancer | Gettinger SN | J. Clin. Oncol. | 2015 | 568 | 86.28 (92) |  |
| 74 | | Nivolumab plus ipilimumab as first-line treatment for advanced non-small-cell lung cancer (CheckMate 012): results of an open-label, phase 1, multicohort study | Hellmann MD | Lancet Oncol. | 2017 | 562 | 112.4 (78) |  |
| 75 | | Safety and Efficacy of Durvalumab (MEDI4736), an Anti-Programmed Cell Death Ligand-1 Immune Checkpoint Inhibitor, in Patients With Advanced Urothelial Bladder Cancer | Segal NH | J. Clin. Oncol. | 2016 | 547 | 102.56 (87) |  |
| 76 | | Combined nivolumab and ipilimumab versus ipilimumab alone in patients with advanced melanoma: 2-year overall survival outcomes in a multicentre, randomised, controlled, phase 2 trial | Hodi FS | Lancet Oncol. | 2016 | 544 | 105.29 (85) |  |
| 77 | | Combined Nivolumab and Ipilimumab in Melanoma Metastatic to the Brain | Tawbi HA | N. Engl. J. Med. | 2018 | 524 | 153.37 (68) |  |
| 78 | | Pembrolizumab versus methotrexate, docetaxel, or cetuximab for recurrent or metastatic head-and-neck squamous cell carcinoma (KEYNOTE-040): a randomised, open-label, phase 3 study | Cohen EEW | Lancet | 2019 | 524 | 174.67 (60) |  |
| 79 | | Updated Analysis of KEYNOTE-024: Pembrolizumab Versus Platinum-Based Chemotherapy for Advanced Non-Small-Cell Lung Cancer With PD-L1 Tumor Proportion Score of 50% or Greater | Reck M | J. Clin. Oncol. | 2019 | 517 | 182.47 (56) |  |
| 80 | | PD-1 Blockade with Cemiplimab in Advanced Cutaneous Squamous-Cell Carcinoma | Migden MR | N. Engl. J. Med. | 2018 | 514 | 146.86 (69) |  |
| 81 | | Efficacy of Pembrolizumab in Patients With Noncolorectal High Microsatellite Instability/Mismatch Repair-Deficient Cancer: Results From the Phase II KEYNOTE-158 Study | Diaz LA | J. Clin. Oncol. | 2020 | 510 | 255 (41) |  |
| 82 | | Pembrolizumab versus paclitaxel for previously treated, advanced gastric or gastro-oesophageal junction cancer (KEYNOTE-061): a randomised, open-label, controlled, phase 3 trial | Shitara K | Lancet | 2018 | 509 | 145.43 (70) |  |
| 83 | | Previous radiotherapy and the clinical activity and toxicity of pembrolizumab in the treatment of non-small-cell lung cancer: a secondary analysis of the KEYNOTE-001 phase 1 trial | Lee P | Lancet Oncol. | 2017 | 488 | 108.44 (83) |  |
| 84 | | Durvalumab plus platinum-etoposide versus platinum-etoposide in first-line treatment of extensive-stage small-cell lung cancer (CASPIAN): a randomised, controlled, open-label, phase 3 trial | Paz-Ares L | Lancet | 2019 | 448 | 206.77 (48) |  |
| 85 | | Pembrolizumab in advanced soft-tissue sarcoma and bone sarcoma (SARC028): a multicentre, two-cohort, single-arm, open-label, phase 2 trial | Tawbi HA | Lancet Oncol. | 2017 | 425 | 102 (88) |  |
| 86 | | Pembrolizumab As Second-Line Therapy in Patients With Advanced Hepatocellular Carcinoma in KEYNOTE-240: A Randomized, Double-Blind, Phase III Trial | Finn RS | J. Clin. Oncol. | 2020 | 422 | 211 (47) |  |
| 87 | | Atezolizumab in combination with carboplatin plus nab-paclitaxel chemotherapy compared with chemotherapy alone as first-line treatment for metastatic non-squamous non-small-cell lung cancer (IMpower130): a multicentre, randomised, open-label, phase 3 trial | Cappuzzo F | Lancet Oncol. | 2019 | 414 | 165.6 (64) |  |
| 88 | | Nivolumab monotherapy in recurrent metastatic urothelial carcinoma (CheckMate 032): a multicentre, open-label, two-stage, multi-arm, phase 1/2 trial | Sharma P | Lancet Oncol. | 2016 | 411 | 79.55 (95) |  |
| 89 | | Combination nivolumab and ipilimumab or nivolumab alone in melanoma brain metastases: a multicentre randomised phase 2 study | Long GV | Lancet Oncol. | 2018 | 408 | 111.27 (79) |  |
| 90 | | Safety and activity of PD1 blockade by pidilizumab in combination with rituximab in patients with relapsed follicular lymphoma: a single group, open-label, phase 2 trial | Neelapu SS | Lancet Oncol. | 2014 | 405 | 50.63 (99) |  |
| 91 | | Atezolizumab plus bevacizumab versus sunitinib in patients with previously untreated metastatic renal cell carcinoma (IMmotion151): a multicentre, open-label, phase 3, randomised controlled trial | Rini BI | Lancet | 2019 | 402 | 155.61 (67) |  |
| 92 | | Safety and antitumour activity of durvalumab plus tremelimumab in non-small-cell lung cancer: a multicentre, phase 1b study | Rizvi NA | Lancet Oncol. | 2016 | 401 | 68.74 (96) |  |
| 93 | | Safety, Efficacy, and Biomarkers of Nivolumab With Vaccine in Ipilimumab-Refractory or -Naive Melanoma | Weber JS | J. Clin. Oncol. | 2013 | 398 | 49.24 (100) |  |
| 94 | | Pembrolizumab for Early Triple-Negative Breast Cancer | Schmid P | N. Engl. J. Med. | 2020 | 396 | 206.61 (49) |  |
| 95 | | Efficacy and Safety of Durvalumab in Locally Advanced or Metastatic Urothelial Carcinoma Updated Results From a Phase 1/2 Open-label Study | Powles T | JAMA Oncol. | 2017 | 382 | 88.15 (91) |  |
| 96 | | Atezolizumab, an Anti-Programmed Death-Ligand 1 Antibody, in Metastatic Renal Cell Carcinoma: Long-Term Safety, Clinical Activity, and Immune Correlates From a Phase Ia Study | McDermott DF | J. Clin. Oncol. | 2016 | 367 | 62.91 (98) |  |
| 97 | | Avelumab, an Anti-Programmed Death-Ligand 1 Antibody, In Patients With Refractory Metastatic Urothelial Carcinoma: Results From a Multicenter, Phase Ib Study | Apolo AB | J. Clin. Oncol. | 2017 | 362 | 80.44 (94) |  |
| 98 | | Nivolumab Versus Docetaxel in Previously Treated Patients With Advanced Non-Small-Cell Lung Cancer: Two-Year Outcomes From Two Randomized, Open-Label, Phase III Trials (CheckMate 017 and CheckMate 057) | Horn L | J. Clin. Oncol. | 2017 | 351 | 85.96 (93) |  |
| 99 | | Five-Year Follow-Up of Nivolumab in Previously Treated Advanced Non-Small-Cell Lung Cancer: Results From the CA209-003 Study | Gettinger S | J. Clin. Oncol. | 2018 | 346 | 96.56 (90) |  |
| 100 | | Programmed Death-1 Blockade With Pembrolizumab in Patients With Classical Hodgkin Lymphoma After Brentuximab Vedotin Failure | Moskowitz CH | J. Clin. Oncol. | 2016 | 341 | 66 (97) |  |
