# Supplementary figures and images for: Research Trends and Most Influential Clinical Studies on Anti-PD1/PDL1 Immunotherapy for Cancers: A Bibliometric Analysis
Source: Front Immunol. 2022 Apr 11;13:862084. doi: 10.3389/fimmu.2022.862084 (PMC9044908; doi:10.3389/fimmu.2022.862084)

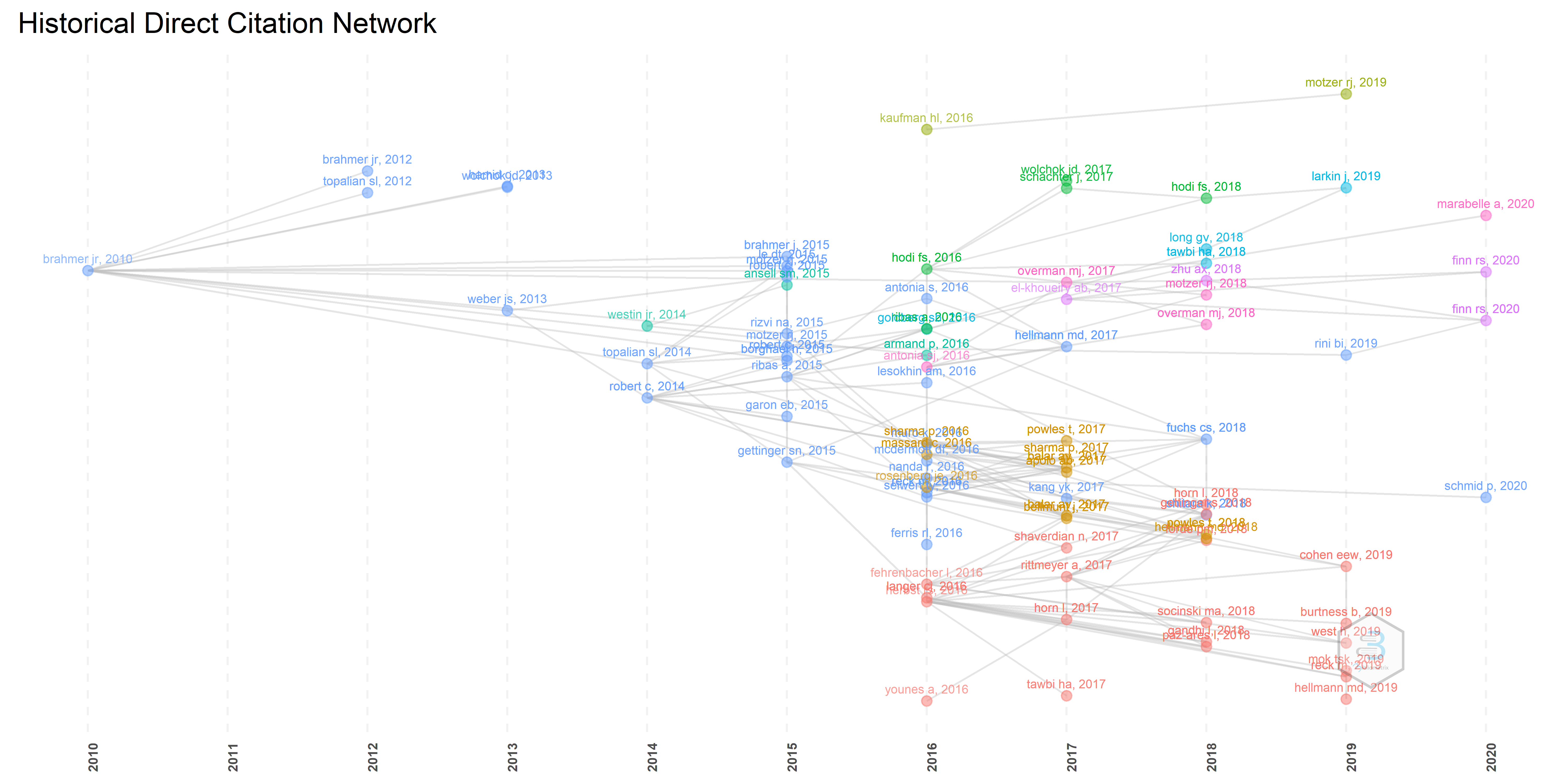

Supplement: Supplementary Figure 1 — Historical direct citation network among the most cited papers. [file Image_1.png]
